# Transdiagnostic group CBT vs. standard group CBT for depression, social anxiety disorder and agoraphobia/panic disorder: Study protocol for a pragmatic, multicenter non-inferiority randomized controlled trial

**DOI:** 10.1186/s12888-016-1175-0

**Published:** 2017-01-23

**Authors:** Sidse M. Arnfred, Ruth Aharoni, Morten Hvenegaard, Stig Poulsen, Bo Bach, Mikkel Arendt, Nicole K. Rosenberg, Nina Reinholt

**Affiliations:** 1 Psychiatric Hospital Slagelse & Psychiatric Research Unit, Region Zealand Psychiatry, Faelledvej 6, Building 3, Level 4., DK-4200 Slagelse, Denmark; 2 Psychotherapeutic Clinic Nannasgade, Mental Health Centre Copenhagen, Capital Region Mental Health Services, Copenhagen University Hospital, Copenhagen, Denmark; 3 Department of Psychology, Faculty of Social Sciences, University of Copenhagen, Copenhagen, Denmark; 4 Unit for Anxiety and Compulsive Disorders, Psychiatric Hospital Risskov, Aarhus University Hospital, Aarhus, Denmark; 5 Institute of Clinical Medicine, Faculty of Health Sciences, University of Copenhagen, Copenhagen, Denmark

**Keywords:** Cognitive Behavior Therapy, Depression, Anxiety, Negative Affect, Clinical Trial, Unified Protocol, Change Mechanisms, Session Tracking

## Abstract

**Background:**

Transdiagnostic Cognitive Behavior Therapy (TCBT) manuals delivered in individual format have been reported to be just as effective as traditional diagnosis specific CBT manuals. We have translated and modified the “The Unified Protocol for Transdiagnostic Treatment of Emotional Disorders” (UP-CBT) for group delivery in Mental Health Service (MHS), and shown effects comparable to traditional CBT in a naturalistic study. As the use of one manual instead of several diagnosis-specific manuals could simplify logistics, reduce waiting time, and increase therapist expertise compared to diagnosis specific CBT, we aim to test the relative efficacy of group UP-CBT and diagnosis specific group CBT.

**Methods/design:**

The study is a partially blinded, pragmatic, non-inferiority, parallel, multi-center randomized controlled trial (RCT) of UP-CBT vs diagnosis specific CBT for Unipolar Depression, Social Anxiety Disorder and Agoraphobia/Panic Disorder. In total, 248 patients are recruited from three regional MHS centers across Denmark and included in two intervention arms.

The primary outcome is patient-ratings of well-being (WHO Well-being Index, WHO-5), secondary outcomes include level of depressive and anxious symptoms, personality variables, emotion regulation, reflective functioning, and social adjustment. Assessments are conducted before and after therapy and at 6 months follow-up. Weekly patient-rated outcomes and group evaluations are collected for every session. Outcome assessors, blind to treatment allocation, will perform the observer-based symptom ratings, and fidelity assessors will monitor manual adherence.

**Discussion:**

The current study will be the first RCT investigating the dissemination of the UP in a MHS setting, the UP delivered in groups, and with depressive patients included. Hence the results are expected to add substantially to the evidence base for rational group psychotherapy in MHS. The planned moderator and mediator analyses could spur new hypotheses about mechanisms of change in psychotherapy and the association between patient characteristics and treatment effect.

**Trial registration:**

Clinicaltrials.gov NCT02954731. Registered 25 October 2016

**Electronic supplementary material:**

The online version of this article (doi:10.1186/s12888-016-1175-0) contains supplementary material, which is available to authorized users.

## Background

Unipolar depression and anxiety disorders are the most prevalent - and often co-occurring - psychiatric disorders in primary health care [1, 2]. These disorders are frequently associated with a chronic, disabling course, functional impairment, and high socio-economic costs [3, 4]. From this perspective, it is imperative to improve mental health service (MHS) treatment programs for these disorders.

Recently, transdiagnostic CBT (TCBT) manuals (e.g.[5, 6]), which employ the same set of treatment principles across several mental disorders (i.e. anxiety disorders and unipolar depression), have been developed to improve the clinical utility of standard diagnosis-specific CBT programs (STD-CBT) [7]. TCBT has demonstrated promising treatment effects comparable to STD-CBT [8, 9]. Potentially, TCBT deals effectively with comorbidity often seen in MHS, reduces waiting time for patients, and reduces training demands and costs for the clinicians. Moreover, principal and comorbid disorders are treated simultaneously, several disorders are treated in the same psychotherapy group, and the clinician only need to be trained in one manual rather than several manuals for different disorders [10, 11].

The “Unified Protocol for Transdiagnostic Treatment of Emotional Disorders” (UP) [12, 13] is one of the most widely studied transdiagnostic manuals [14]. In a recent systematic review and meta-analysis of TCBT for anxiety disorders (including 12 trials, *N* = 1933), TCBT was associated with an overall positive outcome, performed better than waitlist- and treatment as usual comparison interventionsand demonstrated durable treatment gains through follow-up [15]. The pooled treatment effect was moderate (experimental vs control treatment effect size Cohens *d* = .68; [95%CI: 0.45-0.90; *p* < .001) [15]. Large-scale, high quality randomized controlled trials (RCT) are still warranted in order to establish a more solid evidence base concerning the relative efficacy of TCBT and STD-CBT. Individual UP therapy has resulted in reduction of anxiety and depression symptoms for patients with comorbid anxiety disorders in two open trials [16] and one RCT using wait-list comparator [17] as well as one large-scale RCT comparing UP with STD-CBT for anxiety disorders (Barlow 2016, personal communication). Less evidence exists for the effect of UP on depressive disorders, but data from the anxiety trials suggest improvement in comorbid conditions [18]. Limited, but promising, data, including our own naturalistic study suggest that the UP can be delivered in groups with pre-post effect sizes in the medium to large range [19–22]. This is important, as the group format is an efficacious and cost-effective way of delivering treatment [23], which is regularly used in Danish MHS.

Neuroticism/negative affectivity (defined as the tendency to experience frequent and intense negative emotions, including anxiety, fear, anger, sadness, and the like) has been recognized as an important temperamental dimension in major conceptualizations of personality (i.e. the Big Five Model [24]; The Alternative DSM-5 Model for Personality Disorders [25]). Further, findings from recent research suggest that neuroticism is a prominent transdiagnostic factor in the development of emotional disorders [7, 26], which predicts the course of the disorders as well as treatment effect [27]. UP targets neuroticism itself and preliminary findings suggest that negative affectivity is reduced following treatment with the UP [28, 29]. Accordingly, we will investigate whether UP-CBT improves negative affectivity and emotion regulation strategies to a larger extend than STD-CBT.

### Mechanisms of change

To ensure that a treatment effect can be attributed to the hypothesized active ingredients of the specific treatment it is necessary to monitor the fidelity of the implementation of the treatment. Measuring treatment fidelity involves monitoring adherence to a specific treatment manual, assessment of sufficient differentiation between treatment manuals, and assessment of therapist competence, i.e. the level of clinical skills involved in the dissemination of the treatment. Fidelity must be assessed by trained external evaluators, who review a sample of session recordings, and evaluate these three aspects of fidelity by the use of a fidelity rating scale relevant to the specific treatment [30].

Studies suggest that various in-session factors, aside from the specific therapeutic method, contribute to up to 30% of the therapeutic change [31]. We still need a better understanding of what these factors are and how they contribute to treatment outcome [30, 32]. It is already well established that the quality of the therapeutic relationship between the patient and the therapist, and specifically the therapeutic alliance, influence treatment outcome [33, 34]. Likewise, in group psychotherapy relational factors such as group cohesion and a positive group climate are associated with outcome. In STD-CBT the patients have easy access to identification with and understanding of group member’s symptoms. This might be different when the group consists of patients with different diagnoses. Hence, we explore in more detail aspects of the therapeutic relationship between group members.

## Objectives

The main objective is to investigate the effects of group UP-CBT compared with STD-CBT for psychiatric outpatients with a primary diagnosis of Unipolar Depression (DEP), Social Anxiety Disorder (SAD) or Agoraphobia/Panic Disorder (Ag/PD). Main outcomes are subjective well-being, symptom levels, personality traits, emotion regulation, perseverative thinking, and social adjustment. Apart from the primary outcomes, we aim to investigate whether UP-CBT, as proposed, confers changes in negative and positive affectivity and emotion regulation strategies, and, if so, whether these changes are larger in the UP-CBT than in the STD-CBT treatment. Furthermore, we will explore the possible mediating role of treatment factors, including manual adherence and group relational factors, on outcome. Lastly, we will investigate potential moderators of outcome, i.e. patient characteristics like comorbidity, personality traits, reflective function, social network, and adverse life events across and within treatment conditions.

## Hypotheses

We hypothesize that subjective well-being and symptom levels are equally improved following group UP-CBT and group STD-CBT. Based on the treatment target in UP-CBT, we hypothesize that negative affectivity will decrease and emotion regulation strategies will be improved to a higher degree following UP-CBT compared with STD-CBT. We hypothesize that group climate is equivalent in the two arms, and that this and manual adherence mediates a positive outcome. Hypothetical directions of effect of moderators are listed in Table 1.

**Table 1:** Hypothesized effects of moderators in TRACT-RCT

Moderator	UP-CBT> STD-CBT	Outcome^a^
Comorbidity	+	-
Personality Inventory for DSM-5 SF Antagonism	0	-
Personality Inventory for DSM-5 SF Psychoticism	0	-
Personality Inventory for DSM-5 SF Detachment	+	-
Personality Inventory for DSM-5 SF Negative Affect	+	-
Personality Inventory for DSM-5 SF Disinhibition	+	-
Life Event Checklist for DSM-5 (LEC-5)	0	-
Level of Personality Functioning (LPFS-BF)	0	+
Copenhagen Social Relations Questionnaire (CSRQ)^b^	0	+
Reflective Function Questionnaire (RFQ)	-	+

The first colon is read: “when the participants have high levels of the moderator, treatment effect is larger in UP-CBT compared to STD-CBT”. The second colon is read: “when the moderator is high outcome is + (good) or - (bad)”. 0 = no effect/difference between interventions

^a^WHO-5

^b^Positive/supportive relationships

## Methods/design

### Design

**Fig. 1:**
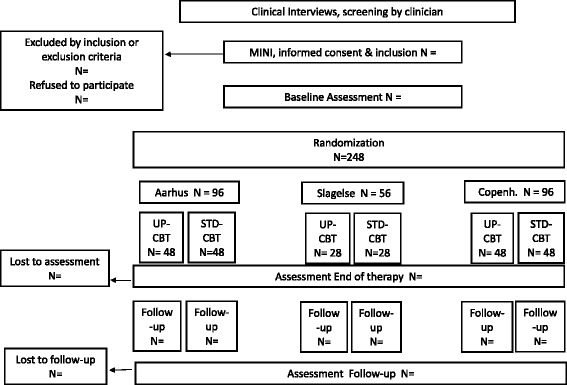
CONSORT Flow diagram TRACT-RCT. UP-CBT: The experimental intervention “Unified Protocol” group CBT. STD-CBT: The comparator intervention standard CBT i.e. diagnosis specific group CBT applying evidence-based protocols

The current trial is an investigator-initiated, partially blinded, pragmatic, parallel, non-inferiority, multi-center randomized clinical trial (RCT). Two equally sized intervention arms, UP-CBT and STD-CBT, are compared. In total, we include 248 patients recruited from three Danish regional MHS. A CONSORT diagram is provided in Fig. 1. Data management is purely digital and is managed by a private enterprise, EasyTrial©, who also assists with randomization, treatment allocation and concealment.

### Settings

The three Danish MHS clinics participating in the study conduct CBT groups for anxiety disorders and/or depression and have previously been engaged in clinical trials. Psychotherapeutic Clinic, Mental Health Centre Copenhagen is located in the inner center of the Capital, the Outpatient Clinics at Risskov Psychiatric Hospital is located in Aarhus, and Psychiatric Outpatient Clinic in Slagelse is located on the isle of Zealand. The number of attending patients and staff is lower at the Slagelse location, hence the sites contribute unequally to the trial. In total, 1400 patients with depression and 550 patients with relevant anxiety disorders attend the participating MHS clinics per year. The clinic in Copenhagen has piloted the UP-CBT as group therapy and two of the researchers have hands-on experience with group UP-CBT and training of UP-CBT therapists for the pilot trial.

### Participants

We aim to include 248 patients that satisfy the inclusion criteria: (1) a principal DSM-5 diagnosis of DEP (single episode or recurrent) (app. 50%), SAD (app. 25%), and Ag/PD (app 25%), (2) age 18-65 years, (3) the patient is currently not using any antidepressants or use accepted antidepressants (according to a predefined protocol, available on request), which have been unchanged for at least 4 weeks before intervention onset and no change in antidepressants is anticipated, (4) sufficient knowledge of the Danish language. Patients will be excluded if (1) risk of suicide is high or moderate according to clinicians or assessment researchers, (2) they have alcohol or drug dependency, (3) they are diagnosed with a cluster A or B (DSM-5) personality disorder by intake clinicians or assessment researcher, (4) they have co-morbidity of pervasive developmental disorder, psychotic disorders, eating disorders, untreated attentional disorder, bipolar disorder, or severe physical illness, (5) they receive psychopharmacological treatments other than those predefined as acceptable, (6) they receive concurrent psychotherapy, (7) they do not accept to stop the use of anxiolytics within the first four weeks of intervention.

### Recruitment procedure and baseline assessment

Patients are typically referred by general practitioners, when they have failed to respond to one or two different treatments (medication and/or psychotherapy). During intake, the patients will be evaluated by clinicians to be eligible for psychotherapeutic treatment in regional MHS as part of the standard procedure in the psychotherapeutic unit and they will be screened for eligibility according to the above described criteria. If patients are eligible for treatment, they will be approached for participation and provided with information about the project. Patients who cannot or will not participate in the study will be offered treatment as usual, i.e. group STD-CBT.

Patients who accept participation, will receive an invitation for further assessment using Mini International Neuropsychiatric Interview ((MINI, v 7.0 for DSM-5) and if eligibility is confirmed, informed consent is acquired. In the same consultation, supplementary baseline ratings and administration of the web-based patient questionnaires on stable patient characteristics, hypothesized to moderate outcome, are performed (see Table 2 for an overview of procedures and time points). Next, after randomization and blinding (see below), within 3 weeks of intervention onset additional baseline ratings are gathered by an outcome assessor making telephone-based interviews and encouraging the patient to answer the web-based outcome assessment questionnaires.

**Table 2:** Overview of procedures and measurements TRACT-RCT

Trial Schedule	Inclusion	Allocation	Post-allocation			
*Time points (weeks)*	*w-3* ^*a*^	*w0*	*w1-2*	*w4-18*	*w19*	*w45*
Enrollment						
MINI	x					
SAPAS	x					
Eligibility screen	x					
Informed consent	x					
Contact details	x					
Web system entrance	x					
Randomization		x				
Intervention arm allocation		x				
CBT group allocation			x			
Interventions						
UP-CBT				x-x		
STD-CBT:						
DEP-CBT				x-x		
SAD-CBT				x-x		
AG/PD-CBT				x-x		
Assessments						
Observer ratings						
Demography	x					
Medical History	x					
Medication History	x				x	x
Hamilton Anxiety Rating Scale HAM-A6			x		x	x
Hamilton Depression Rating Scale HAM-D6			x		x	x
MINI (brief)					x	x
Therapist ratings						
Session Settings				x-x		
Manual Adherence Checklist				x-x		
Group evaluation					x	
Patient questionnaires						
History & Characteristics	x					
Main Outcomes			x	(x) ^b^	x	x
Weekly Outcomes			x	x-x		
Session evaluations				x-x		
Group evaluation					x	
Medicine & Suppl. Treatment					x	x

For detailed overview of patient questionnaires see Table 3, for description of rating scales see text

^a^Inclusion interview will be performed close to patient intake at the clinic and we expect some waiting time before group onset date (not depending on intervention arm) can be established

^b^Primary outcome, WHO-5, is included in both “Weekly outcomes” and “Main outcomes”

### Randomization and blinding

Randomization is performed in blocks of 4 participants, stratified by diagnosis and site, when 16 patients (8 with DEP and 4 with SAD and 4 with Ag/PD) are included (see study flow chart, Fig. 1). EasyTrial © perform the randomization and intervention allocation of study participants. Allocation to experimental intervention or comparison intervention will be computer-generated. In psychotherapy trials, therapists and patients cannot be blinded to intervention type. However, the researcher designated to report the main intervention effects (NR) will be blind to treatment allocation and will not participate in the treatment of study patients. As NR is the only Danish certified UP supervisor she is, however, in charge of UP-CBT training (see below) and continuous supervision of study therapists. The latter will be based on verbal report, and the therapists will be instructed to anonymize the patients in their reports (leaving out name, specific age and other identifiers). EasyTrial© is designed with several access layers, where the data administrators have full access to all data, researchers have access to entered information and treatment allocation, while outcome assessors and NR have limited access, only having lists with names of participants, their timelines and contact details. In this manner, outcome assessors and NR will be blinded to treatment allocation. Furthermore, outcome assessors and NR will not participate in any clinical routines in the clinic. The patients are asked not to discuss their therapy with the outcome assessors and they will not communicate with the therapists. Outcome assessors are asked to guess about treatment allocation after the interviews to be able to analyze possible bias.

For the statistical analyses of intervention effects, intervention type is concealed in the data extraction from EasyTrial©. The analysis of the primary outcome will be conducted with treatment allocation blinded. The intervention and control arm will randomly be coded as “A” and “B” and the deciphering will not be done before conclusions have been formulated based on these codes.

### Interventions

The experimental treatment, group UP-CBT, consists of 8 treatment modules delivered in 14 group sessions given weekly in 2 h sessions. The group manual has been modified from a Danish translation of the published UP for individual therapy [5, 13] and recommendations on group delivery from the UP Institute, Center for Anxiety and Related Disorders (CARD), Boston University. (personal communications) [22].

The SAD STD-CBT manual is based on the main principles described in the individual CBT manual by [35] and the PD/Ago STD-CBT manual is based on the principles described in the individual CBT manual by [36]. The manuals are adapted for use in Danish MHS settings and in groups. The two anxiety manuals are supplemented with frequently used Danish CBT handouts from [37, 38].

The DEP STD-CBT manual is based on a Danish group CBT manual for depression [39]. The manual has previously been used in research studies in Danish MHS [40], and is in line with the interventions described in an American manual [41]. In order to adjust the manual to the settings of group therapy in the TRACT-RCT, an individual group preparation session was added as well as 2 extra group sessions (session 7 and session 12). The work sheets added for these extra sessions are based on [37].

Treatment intensity (number and duration of group sessions) will be equal in both treatment arms. Psychiatric evaluation, extraordinary individual sessions and supplementary occupational counselling or physiotherapy are possible in both intervention arms.

#### Intervention fidelity

In both intervention arms the therapists will be mental health professionals, and in each group at least one therapist will have one-year CBT training and have participated in the pre-trial workshops. UP-CBT therapists attend a two-day UP-CBT workshop conducted by a certified Danish UP therapist (NR). Certification is granted after satisfactory intensive audio-tape supervision of a full pre-trial group course. During the intervention UP-CBT therapists from all sites receive SKYPE©-based monthly group supervision (2 h) by NR. The therapists delivering UP-CBT will not be conducting STD-CBT. STD-CBT-therapists attend a one-day workshop on each of those manuals they will be delivering. The workshops are offered to secure alignment of STD-CBT manuals across sites, and STD-CBT therapists conduct pre-trial STD-CBT groups according to the recommended manuals. During the intervention STD-CBT therapists from each standard group receive SKYPE©-based monthly group supervision (2 h) by a CBT specialist and supervisor. The therapists delivering STD-CBT will not conduct UP-CBT.

In both intervention arms, group sessions will be audio monitored, and random samples (20%) will be rated for adherence to UP-CBT or STD-CBT manual and techniques and therapist competence. For UP fidelity the *Therapist Adherence Rating Scale* (Barlow, personal communication, 2016) is applied. For the STD-CBT groups, manual adherence and therapist competence will be measured by manual specific adherence and competence rating scales, based on, or in case of DEP, inspired by, those previously used by Professor Barlow’s research group [42]. All therapists also carry out self-monitoring of CBT techniques as they check a list of specific interventions after each group session (see below). Additionally, information regarding non-specific program adherence such as waiting time, stability of therapists, session cancellation, delay etc. will be collected at each session.

### Assessment

Data are gathered through a number of ratings from patients, therapists and observers prior to inclusion, weekly throughout the intervention period, at the end of therapy, and at 6 months follow-up. An overview of assessment procedures is given in Table 2, and instruments in each category are detailed below. Outcome assessors are medical or psychology students, who will be certified in the use of relevant rating scales and will be trained to excellent agreement on the observer instruments by experienced psychotherapy researchers prior to inclusion start and with continuous monthly co-ratings and reliability assessments based on audiotaped interviews. Main outcomes are collected by outcome assessors along with patient self-ratings at end of the intervention and at 6 months follow-up. Every week, patients and therapists will fill out brief questionnaires immediately before and after the group session. If participants discontinue the intervention, we will attempt to gather all data, except those relating to intervention participation (session ratings, therapy evaluation), unless the patient explicitly denies participating in data collection (Table 3).

**Table 3:** Patient Questionnaires

Questionnaires for Patients	Inclusion	Allocation	Baseline	Group therapy	End of group	Follow-up
*Timepoint (week number)*	*w-3*	*w0*	*w1-2*	*w4-18*	*w19*	*w45*
History & Characteristics						
Personality Inventory for DSM-5 Short Form (PID-5 SF)	x					
Level of Personality Functioning – Brief Form (LPFS-BF)	x					
Life Event Checklist for DSM-5 (LEC-5)	x					
Copenhagen Social Relations Questionnaire (CSRQ)	x					x
Reflective Function Questionnaire (RFQ-54)	x					
Main Outcomes						
WHO Well-Being Index (WHO-5)			x	x-x	x	x
Hopkins Symptom Check List (SCL-25)			x		x	x
Positive Affect and Negative Affect Schedule (PANAS)			x		x	x
Emotion Regulation Questionnaire (ERQ)			x		x	x
Emotion Regulation Strategies Questionnaire (ERSQ)			x		x	x
Perserverative Thinking Questionnaire (PTQ)			x		x	x
Becks Depression inventory (BDI-II)			x		x	x
Work and Social Adjustment Scale (WSAS)			x		x	x
Personality Inventory for DSM-5 (PID-5-SF) Internalizing Features					x	x
Client Satisfaction Questionnaire (CSQ)					x	
*Depending on primary anxiety diagnosis:*						
Panic Disorder: Panic Disorder Severity Scale (PDSS)			x		x	x
Social Anxiety Disorder: Liebowitch Social Anxiety Scale (LSAS)			x		x	x
Agoraphobia: Mobility Inventory for Agoraphobia (MIA)			x		x	x
Weekly Outcomes						
Overall Anxiety Severity and Impairment Scale (OASIS)			x	x-x		
Overall Depression Severity and Impairment Scale (ODSIS)			x	x-x		
Homework Tracker (HWT)				x-x		
Session evaluations						
Group Questionnaire 12-items (GQ12)				x-x		
Group evaluation						
Evaluation of the course of therapy					x	
Medicine & Suppl Treatment						
Purpose-made questions	x				x	x

### Primary outcome

#### Who Well-Being Index (WHO-5)

The primary outcome is the self-rating instrument WHO Well-Being Index, 5 items (WHO-5). It is tracked weekly during treatment, at the end of treatment and at follow-up. It is a short questionnaire used to access subjective well-being. It is derived from a 28-item Psychological Well-Being Schedule, where the five items indicating general well-being produced a unidimensional positive dimension of well-being. The raw scores are transformed to a score from 0 (worst thinkable well-being) to a 100 (best thinkable well-being). A score <50 suggests poor emotional well-being and scores ≤28 indicate depression [43]. The WHO-5 Well-being Index has demonstrated high reliability, validity and sensitivity to treatment response for affective and neurotic disorders in psychiatric care [44, 45]. It is considered a very sensitive outcome measure as it does not incorporate negative quality of life, i.e. distress, and has no ceiling effect [46].

### Secondary outcomes

#### Observer ratings

These ratings are performed by researchers (clinical psychologists) at baseline and by outcome assessors at later time points. The telephone based outcome assessment will also provide opportunity for coaching the patient to fill out self-report questionnaires.

##### Mini International Neuropsychiatric Interview (mini)

MINI was developed by Sheehan and Lecrubier in 1992. MINI, v. 7.0 for DSM-5 will be applied. Version 7.0 contains 17 modules for the major axis I psychiatric disorders in DSM-IV-TR and ICD-10. MINI is validated against SCID-P for DSM-IV and CIDI for ICD-10 [47]. Psychometric analyses of the MINI have demonstrated acceptable test-retest and inter-rater reliability. MINI is divided into modules identified by letters (A-P), each corresponding to a diagnostic category. At the enrolment interview we include all modules but P, and use the diagnostic assessment for eligibility evaluation and description of comorbidity. At end of therapy and follow-up we only use modules A, D, E and F for assessment of recovery or relapse of primary diagnoses.

##### Standardized Assessment of Personality – Abbreviated Scale (SAPAS)

MINI assessment will be supplemented with SAPAS as a screening for comorbid personality problems. It consists of eight items scoring 0 or 1, summing up to a maximum of 8. A score of 3 and above has been shown to predict DSM-IV personality disorder diagnosis [48].

##### Hamilton Anxiety Rating Scale HAM-A6

HAM-A6 is a focused version of the original 14-item Hamilton Anxiety Scale (HAM-A) [49]. It covers six symptoms of anxiety disorders as one homogenous factor (total score) with high discriminant validity in outcome studies [50–52].

##### Hamilton Depression Rating Scale HAM-D6

HAM-D6 is based on the original 17-item Hamilton Depression Rating Scale [53]. Item analyses have shown that the six included items validly reflect a global depression assessment by experts and a total HAM-17 score [54, 55].

#### Patient self-ratings

The secondary patient rated outcomes are measured at baseline, at end of treatment and at follow-up. Three scales are only presented for those patients with the diagnosis: PD: Panic Disorder Severity Scale (PDSS); Ag: Mobility Inventory for Agoraphobia (MIA); SAD: Liebowitz Social Anxiety Scale – Self-Report (LSAS-SR).

##### Hopkins Symptom Checklist (SCL-25)

SCL-25 is a widely-used questionnaire of self-reported psychological symptoms and psychological distress, which has been used in clinical screening and outcome research [56, 57]. It exists in several formats from 6 to 92 items. In the current study, one of the original brief versions consisting of 25 items, tracking two of the original nine symptom dimensions (depression and anxiety) is used [58].

##### The Positive and Negative Affect Schedule (PANAS)

PANAS [59] is included as a secondary outcome measure of negative and positive affect. PANAS is a brief, valid, and reliable self-report measure of core negative affect and deficits in positive affect. It consists of 20 emotion words, which are rated on a scale from 0 (very slightly or not at all) to 5 (extremely), indicating the extent to which the patient experienced the emotion past week. PANAS has shown high internal consistency, stability and validity [60]. The subscales also showed acceptable to good internal consistencies (negative affect: *α* = .78; positive affect: *α* = .85).

##### Emotion Regulation Questionnaire (ERQ)

ERQ is a 10-item instrument, which assesses the typical use of two general emotion regulation strategies: emotion suppression (i.e. ‘I keep my emotions to myself’) and reappraisal (i.e. “When I want to feel less negative emotion, I change the way I'm thinking about the situation”). The items are rated on a 7-point Likert scale from 1 (strongly disagree) to 7 (strongly agree). The scale has been shown to possess good psychometric properties [61].

##### Emotion Regulation Strategies Questionnaire (ERSQ)

ERSQ is a 27-item self-report instrument that measures the use of emotion-regulation skills during the previous week on a five-point Likert scale (“not at all” to “almost always”). The questionnaire includes nine scales measuring different emotion regulation skills (i.e. ‘I paid attention to my feelings’; ‘I was able to accept my negative feelings’). The instrument possesses good internal consistency (*α* = .90), adequate retest-reliability (*r* = .75) as well as sensitivity to change [62]. All subscales have demonstrated convergent and discriminate validity, including strong positive correlations with constructs related to emotion regulation.

##### Perserverative Thinking Questionnaire (PTQ)

PTQ is a 15-item questionnaire. It was developed to measure repetitive negative thinking, [63] which has been found to be involved in the maintenance of emotional problems, i.e. anxiety and depression [64]. Participants rate how they typically think about negative experiences or problems. Scores range from 0 to 60 and higher scores indicate more frequent engagement in repetitive negative thinking. PTQ has shown excellent internal consistency and correlate well with other measure of repetitive negative thinking, e.g. The Penn State Worry Questionnaire [63].

##### Beck Depression Inventory-II (BDI)

BDI was developed for quantification of depressive symptoms in patients diagnosed with major depression according to DSM-IV [65]. It has been widely used internationally and correlates well with clinical evaluations and observer ratings of depression like the Hamilton Depression Rating Scale [66]. The inventory addresses the patient’s mood and behaviour over the previous two weeks. Scores range from 0 to 63; 0-13 indicates minimal depression, 14-19 mild depression, 20-28 moderate depression, and 29-63 severe depression [65].

##### Work and Social Adjustment Scale (WSAS)

Work and Social Adjustment Scale (WSAS) [67] represents a simple measurement of impairment of functioning. It consists of five items, each rated on an 8-point severity scale adding up to a maximum severity of 40 points. It is validated for use across the full spectrum of psychiatric disorders and is used in epidemiological research as well as clinically [68].

##### Client Satisfaction Questionnaire (CSQ)

CSQ is an 8-item scale loading to one factor of satisfaction with mental health care service. Responses are 1-4, where 1 is “very or definitely dissatisfied and 4 is “very or definitely satisfied.” It does not include a neutral rating [69, 70]. Scoring was originally reported as a sum score 8-32. A Danish translation has been widely used in the mental health service, but it has not been validated.

##### Panic Disorder Severity Scale (PDSS)

PDSS is a brief rating scale with seven items assessing the severity of PD symptoms and functional impairment. Items are rated on a 0- to 4-point scale [71, 72].

##### Liebowitz Social Anxiety Scale - Self-Report (LSAS-SR)

LSAS-SR assesses a range of situations typically feared by individuals with social anxiety. It has 24 items, divided into subscales of performance anxiety and social situations. It has acceptable psychometric properties and the self-report version provides scores close to the original observer rating [73].

##### Mobility Inventory for Agoraphobia (MIA)

MIA assesses agoraphobic avoidance. It consists of 27 items rated on a 1-5 point scale for avoidance. Each situation is described with the patient accompanied or alone, resulting in two different measures of avoidance [74].

### Exploratory measures

#### History and moderating characteristics

##### Level of Personality Functioning - Brief Form 2.0 (LPFS-BF)

LPFS-BF is a brief 12-item self-report inventory developed to assess levels of personality functioning as defined in the alternative model for personality disorders in DSM-5 Section III [75–77]. The LPFS-BF measures impairment in personality functioning within the domains of self-functioning (e.g., Identity and Self-direction) and interpersonal functioning (e.g., Empathy, Self-esteem, and Intimacy). Such features of functioning are considered essential in adapting to (stressful) life events. [78].

##### Reflective Functioning Questionnaire (RFQ)

RFQ is a 54-item self-report inventory assessing the respondent’s ability to understand the internal mental states of self and others. Items are phrased as statements, and the response options are 1-6 (from totally disagree to totally agree). It captures the mentalizing capacity in two domains: Certainty or Uncertainty about mental states, and low scores are associated with low level personality function [79, 80].

##### Personality Inventory for Dsm-5 - Short Form (PID-5 SF)

PID-5 SF isan abbreviated 100-item version of the Personality Inventory for DSM-5 (PID-5) developed to measure the pathological trait specifiers listed in the alternative model for personality disorders in DSM-5 Section III [76, 81]. The PID-5-SF describes 25 trait facets organized in five higher-order domains: Negative Affectivity (vs. Emotional Stability), Detachment (vs. Extraversion), Antagonism (vs. Aggreableness), Disinhibition (vs Conscientiousness), and Psychoticism (vs. Lucidity). The Danish PID-5-SF has demonstrated sound psychometric qualities [82, 83]. In the follow-up part of the present trial, we use composite scores of internalizing personality features (i.e., Negative Affectivity and Detachment domain scores) derived from the PID-5 SF.

##### Life Event Checklist for DSM-5 (LEC-5)

LEC-5 is a self-report measure designed to screen for potentially traumatic events in the patient's lifetime. The LEC-5 assesses exposure to 17 types of events known to potentially result in distress. Response options are “happened to me”, “witnessed it” “learned about it” “part of my job” “not sure” and “doesn’t apply”. LEC-5 has demonstrated adequate psychometric properties as a stand-alone assessment of traumatic exposure [84].

##### Copenhagen Social Relations Questionnaire (CSRQ)

CSRQ is used for the assessment of the structure and function of the patient’s social network. It consists of 8 questions pertaining to 5 domains (i.e. parents, friends, family, children or neighbor’s) and the response options are ‘always’, ‘often’, ‘sometimes’, ‘seldom’, ‘never’, ‘have none’. It was developed for and validated in a Danish middle-aged population [85, 86].

#### Weekly outcomes – session tracking

##### Overall Anxiety Severity and Impairment Scale (OASIS)

OASIS is a five-item self-report instrument of severity of and impairment due to anxiety with established concurrent and discriminant validity. It addresses anxiety frequency and severity, level of avoidance, work/school/home interference, and social interference on a five-point Likert scale [87, 88].

##### Overall Depression Severity and Impairment Scale (ODSIS)

ODSIS is a five-item self-report measure of severity of and impairment due to depression, with established concurrent and discriminant validity. The scale addresses depression frequency and severity, level of engagement, work/school/home interference, and social interference on a five-point Likert scale [89].

##### Home-Work Tracker (HWT)

HWT is purpose-made for this study. It is questions concerning patients’ adherence to home-work as well as symptom-coping during the preceding week.

#### Session evaluations

##### Group Questionnaire - Brief 12-Items (GQ12)

The original Group Questionnaire is a 30 item, 0-7 Likert scale, patient self-rating, session to session questionnaire with a well-established construct validity, measuring 3 different relational domains in group therapy: positive bonding, positive working, and negative relationships [90]. In the current study we will use the recently developed 12 item Group Questionnaire (Burlingame, personal communication, 2016).

#### Evaluation of the course of therapy

A questionnaire focusing on the patient’s evaluation of the group therapy, which was purpose-made for the current study, will be distributed at the end of the group course. Items have been constructed as Likert Scale feedback forms consisting of a list of statements about different aspects of the course of group therapy. Response possibilities are five categories ranging from very much in agreement to not at all.

#### Therapist ratings

##### Manual adherence checklist

A purpose-made self-evaluation check-list for therapists, designed as a list of possible interventions will be employed. At the end of each session, therapists report to which degree they used each intervention from the list. The results will be analyzed according to a pre-defined algorithm, defining optimal UP and TAU-CBT interventions at different stages of treatment, and discriminating between allowed and prohibited interventions. The intervention check-list will be compared to the external fidelity assessment.

##### Session setting

A fact sheet about session cancellation, change in therapists, attendance, change of group room or session length has been developed specifically for the study.

### Statistical considerations

Sample size estimation is based on the primary outcome measure, WHO-5. In our initial naturalistic study of group UP, pre-treatment WHO-5 was 34.5 (SD 17.3) and end-of-treatment 44.8 (SD 17.4) (N = 46) (22). WHO-5 has a minimum clinical significant difference of 10 (Bech, personal communication, 2016). For non-inferiority trials, it is paramount to select a relevant limit for the possible difference in WHO-5 score that will lead to rejection of the hypothesis of non-inferiority [91]. We have set the limit to 9, i.e., one point below the minimum clinical significant difference. Alpha is set to 5% to avoid type I error, resulting in a 95% one-sided confidence interval for decision of non-inferiority. Beta is generally set to 0.1, i.e. a power of 90%. Applying end-point WHO-5 SD of 17,4 and limit of difference delta: 9 points, if UP-CBT is no less effective than TAU-CBT, 204 patients are needed (102 in each arm) to be 90% sure that the lower limit of a 95% one-sided confidence interval will be above the non-inferiority limit of - 9. Meeting the risk of drop-outs with an increase of 20% we will include 124 participants in each arm, in total 248 patients.

Main outcomes will be analyzed in an intention-to-treat (ITT) design, where we will use multiple imputations to handle missing data. All primary analyses will be adjusted for the stratification variables (site, diagnosis). We will use mixed regression analyses that will enable us to use all outcome ratings and enter separate levels for groups, therapists and clinical sites. For exploratory mediator analyses, we will use per-protocol data as well as ITT and both Autoregressive Cross-Lagged Modeling and Structural Equation Modeling [92].

### Ethics and governance

The trial will be conducted according to national and international standards of good clinical practice. All participants will be provided with written and verbal information on the trial so that they can make an informed decision about their participation. Data will be collected and handled confidentially in accordance with the rules of the Danish Data Protection Agency. The protocol has been approved by the Ethics Committee Region Zealand (Registration number: 3084871-SJ-582) and the Danish Data Protection Agency Region Zealand (Registration number: REG-104-2016). Protocol amendments will be communicated at https://clinicaltrials.gov and detailed in publications. STD-CBT is considered an appropriate and ethically justified control group, as it is the recommended treatment for the target group prescribed by the Regional and National treatment programs in Denmark.

Waiting time to treatment may be slightly increased due to the project, as the UP-CBT groups reduce the number of patients available for the STD-CBT groups, but it is not expected to increase above the limit of the clinically acceptable.

We monitor for adverse events, in particular suicidal behavior/ideation. Change of medication or withdrawal from the study will be considered in case of increased suicidal ideation/behavior. First step of change in medication will, if possible, be dose increase of already instituted treatment. Next step will be to initiate or cross over to another type of medication according to a predefined protocol (available on request) established in accordance with the clinical guidelines in the participating clinics and the Danish Health Authorities. In case the responsible psychiatrist considers it necessary to initiate treatment with hypnotics or anxiolytics, it is possible to do so, within a limited dose range and according to a predefined protocol (ibid). If it is necessary to institute medical treatment, not listed in the predefined guidelines, the patient will continue in group therapy and in the trial, but the incident will be recorded as an important change of medicine and as an adverse event.

The first author, SMA, is principal investigator (PI) and initiated the project. Following a formal collaboration agreement between the participating regions, Region Zealand owns the data, and PI has the right to extract and distribute data to participating researchers. A steering committee consisting of the heads of the participating units, ensures the integration of the project with the day-to-day running of the units, while the scientific project group consisting of the authors of this protocol, headed by the PI, is in charge of scientific decisions related to design, data collection, data analysis and reporting and conducting the study in line with Good Clinical Practice guidelines. Signed contracts describe the rights of the involved young researchers as to writing up manuscripts and authorships. The study does not have a data monitoring committee, as allocation is not concealed for therapists.

## Discussion

The current study will be the first RCT investigating the dissemination of the UP in a MHS setting. Also the current study will be the first RCT on the UP delivered in groups. There is a potential risk that the patients in UP may not be able to recognize their problems in the symptoms targeted in the group therapy and may, accordingly, have an increased risk of drop out or of experiencing no effect of the therapy. It is furthermore possible that being in a group with patients with quite different symptoms might be experienced as irrelevant or even overwhelming by the patients. The sensitivity for these aspects of UP might differ between diagnostic groups i.e. resulting in patients with SAD perhaps having less effect of UP, than the other patients. Lack of group cohesion may ultimately lead to non-attendance and drop-out of treatment and accordingly to long-term negative outcome i.e. chronic or recurrent symptoms [93]. Consequently, it is important to have weekly symptom and attendance tracking. Many of the problems mentioned may, however, also be seen in STD-CBT. The risk of not having adequate benefit from STD-CBT is also high as the number of patients needed to treat for the addition of one more remitted patient is four, when comparing CBT for depression with no intervention [94]. These numbers make the introduction of new treatments highly relevant; we need to improve CBT beyond what has been established as efficient, or to find out how to individualize treatment strategies.

If our results support the application of UP group therapy in MHS, we expect that it will be much more feasible to implement group therapy for depression and anxiety disorders in smaller clinics, within MHS outside the main cities and in the primary sector. At this point, even at the university hospitals, we see quite lengthy waiting time before group therapy commences. At the same time, the application of only one manual will support focused training and supervision, hence increasing the expertise of therapists. In larger clinics, the use of UP could also make it possible to compose groups based on other characteristics than diagnoses i.e. gender, age or life events. Lastly, the use of UP for those patients, frequently seen in the MHS, with comorbidity in the form of two equally important disorders might be particularly advantageous.

By virtue of the large-scale, randomized design, the current study will add to the preliminary results of TCBT, providing information on the efficacy of TCBT compared to STD-CBT. The explorative moderation and mediation analyses could spur new hypotheses about mechanisms of change and the association between treatment effect and patient characteristics. Future research might focus on possible improvements of the UP manual, and a particular focus on those patients who might not benefit from either UP-CBT or STD-CBT.

### Trial status

Training of therapists and outcome assessors is ongoing. Recruitment commences December 2016 and will continue until September 2018.

## Additional file

Additional file 1: SPIRIT 2013 Checklist: Recommended items to address in a clinical trial protocol and related documents. (DOC 122 kb)

## Abbreviations

CBT: Cognitive Behavior Therapy
MHS: Mental Health Service
RCT: Randomized Controlled Trial
STD-CBT: Standard CBT i.e. diagnosis specific manualized CBT
T-CBT: Transdiagnostic CBT
UP: the “Unified Protocol for Transdiagnostic Treatment of Emotional Disorders” – a specific manual for transdiagnostic CBT [5, 13]
UP-CBT: The revised Danish group version of the Unified Protocol for Transdiagnostic Treatment of Emotional Disorders [22]

## References

[1] Kessler RC, Berglund P, Demler O, Jin R, Merikangas KR, Walters EE (2005). Lifetime prevalence and age-of-onset distributions of DSM-IV disorders in the National Comorbidity Survey Replication. Arch Gen Psychiatry.

[2] Brown TA, Campbell LA, Lehman CL, Grisham JR, Mancill RB (2001). Current and lifetime comorbidity of the DSM-IV anxiety and mood disorders in a large clinical sample. J Abnorm Psychol.

[3] Goldberg DP, Krueger RF, Andrews G, Hobbs MJ (2009). Emotional disorders: cluster 4 of the proposed meta-structure for DSM-V and ICD-11. Psychol Med.

[4] Kessler RC, Chiu WT, Demler O, Merikangas KR, Walters EE (2005). Prevalence, severity, and comorbidity of 12-month DSM-IV disorders in the National Comorbidity Survey Replication. Arch Gen Psychiatry.

[5] Barlow DH, Farchione TJ, Fairholme CP, Ellard KK, Boisseau CL, Allen LB, Ehrenreich-May JT (2011). Unified Protocol for Transdiagnostic Treatment of Emotional Disorders: Therapist Guide.

[6] Norton PJ (2012). A randomized clinical trial of transdiagnostic cognitve-behavioral treatments for anxiety disorder by comparison to relaxation training. Behav Ther.

[7] Barlow DH (2004). Toward a Unified Treatment for Emotional Disorders. Behav Ther.

[8] McEvoy P, Nathan P, Norton P (2009). Efficay of transdiagnostic treatments: A reveiw of published outcome studes and future research directions. J Cogn Psychother.

[9] Norton PJ (2008). An open trial of a transdiagnostic cognitive-behavioral group therapy for anxiety disorder. Behav Ther.

[10] McManus F, Shafran R, Cooper Z (2010). What does a transdiagnostic approach have to offer the treatment of anxiety disorders?. Br J Clin Psychol.

[11] Wilamowska ZA, Thompson-Hollands J, Fairholme CP, Ellard KK, Farchione TJ, Barlow DH (2010). Conceptual background, development, and preliminary data from the unified protocol for transdiagnostic treatment of emotional disorders. Depress Anxiety.

[12] Barlow DH (2011). Unified protocol for transdiagnostic treatment of emotional disorders : therapist guide.

[13] Barlow DH, Ellard KK, Fairholme CP, Farchione TJ, Boisseau CL, Ehrenreich-May JT (2011). Unified Protocol for Transdiagnostic Treatment of Emotional Disorders: Work Book.

[14] Norton PJ, Paulus DJ. Toward a Unified Treatment for Emotional Disorders: Update on the Science and Practice. Behavior Therapy. 2015 in press. 10.1016/j.beth.2015.07.002 27993337

[15] Reinholt N, Krogh J (2014). Efficacy of transdiagnostic cognitive behaviour therapy for anxiety disorders: a systematic review and meta-analysis of published outcome studies. Cogn Behav Ther.

[16] Ellard KK, Fairholme CP, Boisseau CL, Farchione TJ, Barlow DH (2010). Unified Protocol for the Transdiagnostic Treatment of Emotional Disorders: Protocol Development and Initial outcome data. Cogn Behav Pract.

[17] Farchione TJ, Fairholme CP, Ellard KK, Boisseau CL, Thompson-Hollands J, Carl JR, Gallagher MW, Barlow DH (2012). Unified protocol for transdiagnostic treatment of emotional disorders: a randomized controlled trial. Behav Ther.

[18] Davis L, Barlow DH, Smith L (2010). Comorbidity and the treatment of principal anxiety disorders in a naturalistic sample. Behav Ther.

[19] de Ornelas Maia AC, Nardi AE, Cardoso A (2015). The utilization of unified protocols in behavioral cognitive therapy in transdiagnostic group subjects: A clinical trial. J Affect Disord.

[20] Bullis JR, Sauer-Zavala S, Bentley KH, Thompson-Hollands J, Carl JR, Barlow DH (2015). The unified protocol for transdiagnostic treatment of emotional disorders: preliminary exploration of effectiveness for group delivery. Behav Modif.

[21] Sauer-Zavala S, Bentley KH, Wilner JG (2016). Transdiagnostic Treatment of Borderline Personality Disorder and Comorbid Disorders: A Clinical Replication Series. J Pers Disord.

[22] Reinholt N, Aharoni R, Winding C, K. RN, Rosenbaum B, Arnfred SM. Transdiagnostic Group CBT for Anxiety Disorders: The Unified Protocol in Mental Health Services. Cognitive Behavior Therapy. 2016, in press. 10.1080/16506073.2016.1227360 27705086

[23] McDermut W, Miller IW, Brown RA (2001). The efficacy of group therapy for depression: A meta-analysis and review of the empirical research. Clinical Psychology: Science and Practice.

[24] Digman (1990). Personality Structure: Emergence of the five-factor model. Annu Rev Psychol.

[25] APA (1994). Diagnostic and Statistical Manual of Mental Disorders.

[26] Brown TA, Chorpita BF, Barlow DH (1998). Structural relationships among dimensions of the DSM-IV anxiety and mood disorders and dimensions of negative affect, positive affect, and autonomic arousal. J Abnorm Psychol.

[27] Brown TA (2007). Temporal course and structural relationships among dimensions of temperament and DSM-IV anxiety and mood disorder constructs. J Abnorm Psychol.

[28] Carl JR, Gallagher MW, Sauer-Zavala SE, Bentley KH, Barlow DH (2014). A preliminary investigation of the effects of the unified protocol on temperament. Compr Psychiatry.

[29] Sauer-Zavala S, Boswell JF, Gallagher MW, Bentley KH, Ametaj A, Barlow DH (2012). The role of negative affectivity and negative reactivity to emotions in predicting outcomes in the unified protocol for the transdiagnostic treatment of emotional disorders. Behav Res Ther.

[30] Dobson KS, Singer AR (2005). Definitional and Practical Issues in the Assessment of Treatment Integrety. Clinical Psychology: Science and Practice.

[31] Lambert MJ, Barley DF (2001). Research summary on the therapeutic relationship and psychotherapy outcome. Psychotherapy: Theory, Research, Practice, Training.

[32] Kazdin AE (2005). Treatment outcomes, common factors and continued neclect of mechanisms of change. Clinical Psychology: Science and Practice.

[33] Zilcha-Mano S, Muran JC, Hungr C, Eubanks CF, Safran JD, Winston A (2016). The relationship between alliance and outcome: Analysis of a two-person perspective on alliance and session outcome. J Consult Clin Psychol.

[34] Zilcha-Mano S, Snyder J, Silberschatz G. The effect of congruence in patient and therapist alliance on patient's symptomatic levels. Psychother Res. 2016;1–10. 10.1080/10503307.2015.1126682 26837661

[35] Turk C, Heimberg R, Magee L, Barlow DH (2008). Social Anxiety Disorder. Clinical handbook of psychological disorders.

[36] Craske MG, Barlow D, Barlow DH (2008). Panic Disorder and Agoraphobia. Clinical handbook of psychological disorders.

[37] Arendt, Rosenberg. Kognitiv terapi, 1. udgave edn. Kbh.: Hans Reitzel; 2012.

[38] Suhl S, Arpe-Moeller C, Gravesen L (2016). Gruppemanual for panikangst og agorafobi: 2. og 3. bølge kognitiv adfærdsterapi.

[39] Due Madsen J (2008). Kognitiv adfærdsterapi ved depression.

[40] Lau ME, Calmer J, Bramsen J, Madsen JD (2008). Short-term treatment of depression. Implementation of group-based cognitive behavioral therapy. Ugeskr Laeger.

[41] Scott MJ (2011). Simply effective group cognitive behaviour therapy : a practitioner's guide.

[42] Boswell JF, Gallagher MW, Sauer-Zavala SE, Bullis J, Gorman JM, Shear MK, Woods S, Barlow DH (2013). Patient characteristics and variability in adherence and competence in cognitive-behavioral therapy for panic disorder. J Consult Clin Psychol.

[43] Lowe B, Spitzer RL, Grafe K, Kroenke K, Quenter A, Zipfel S, Buchholz C, Witte S, Herzog W (2004). Comparative validity of three screening questionnaires for DSM-IV depressive disorders and physicians' diagnoses. J Affect Disord.

[44] Newnham EA, Hooke GR, Page AC (2010). Monitoring treatment response and outcomes using the World Health Organization's Wellbeing Index in psychiatric care. J Affect Disord.

[45] Newnham EA, Hooke GR, Page AC (2010). Progress monitoring and feedback in psychiatric care reduces depressive symptoms. J Affect Disord.

[46] Bech P, Olsen LR, Kjoller M, Rasmussen NK (2003). Measuring well-being rather than the absence of distress symptoms: a comparison of the SF-36 Mental Health subscale and the WHO-Five Well-Being Scale. Int J Methods Psychiatr Res.

[47] Sheehan DV, Lecrubier Y, Sheehan KH, Amorim P, Janavs J, Weiller E, Hergueta T, Baker R, Dunbar G (1998). The Mini-International Neuropsychiatric Interview (M.I.N.I.): the development and validation of a structured diagnostic psychiatric interview for DSM-IV and ICD-10. J Clin Psychiatry.

[48] Moran P, Leese M, Lee T, Walters P, Thornicroft G, Mann A (2003). Standardised Assessment of Personality - Abbreviated Scale (SAPAS): preliminary validation of a brief screen for personality disorder. Br J Psychiatry.

[49] Hamilton M (1959). The assessment of anxiety states by rating. British Journal of Medical Psychology.

[50] Bech P (2007). Dose-response relationship of pregabalin in patients with generalized anxiety disorder. A pooled analysis of four placebo-controlled trials. Pharmacol Toxicol.

[51] Meoni P, Salinas E, Brault Y, Hackett D (2001). Pattern of symptom improvement following treatment with venlafaxine XR in patients with generalized anxiety disorder. J Clin Psychiatry.

[52] Bech, Psykiatrisk Hospital i Hillerød (2005). Rating scales for affektive lidelser [Rating scales for Affective Disorders].

[53] Hamilton M (1960). A rating scale for depression. J Neurol Neurosurg Psychiatry.

[54] Bech P, Gram LF, Dein E, Jacobsen O, Vitger J, Bolwig TG (1975). Quantitative rating of depressive states. Acta Psychiatr Scand.

[55] Bech P (2006). Rating scales in depression: limitations and pitfalls. Dialogues Clin Neurosci.

[56] Derogatis LR, Lipman RS, Rickels K, Uhlenhuth EH, Covi L (1974). The Hopkins Symptom Checklist (HSCL): a self-report symptom inventory. Behav Sci.

[57] Lipman RS, Covi L, Shapiro AK (1979). The Hopkins Symptom Checklist (HSCL)--factors derived from the HSCL-90. J Affect Disord.

[58] Müller J, Poster C, Beyer T, Furniss T, Actergarde S (2010). Comparison of Eleven Short Versions of the Symptom Checklist 90-Revised (SCL-90-R) in the assessment of general Psychopathology. J Psychopathol Behav Assess.

[59] Watson D, Clark LA, Tellegen A (1988). Development and validation of brief measures of positive and negative affect: The PANAS scales. J Pers Soc Psychol.

[60] Krohne HW, Egloff B, Kohlmann CW, Tausch A (1996). Positive and Negative Affect Schedule (PANAS). Diagnostica.

[61] Gross JJ, John OP (2003). Individual differences in two emotion regulation processes: implications for affect, relationships, and well-being. J Pers Soc Psychol.

[62] Berking M, Wupperman P, Reichardt A, Pejic T, Dippel A, Znoj H (2008). Emotion-regulation skills as a treatment target in psychotherapy. Behav Res Ther.

[63] Ehring T, Zetsche U, Weidacker K, Wahl K, Schonfeld S, Ehlers A (2011). The Perseverative Thinking Questionnaire (PTQ): validation of a content-independent measure of repetitive negative thinking. J Behav Ther Exp Psychiatry.

[64] Ehring T, Watkins E (2008). Repetitive Negative Thinking as a Transdiagnostic Process. Int J Cogn Ther.

[65] Beck AT, Steer RA, Brown GK (1996). Manual for the BDI.

[66] Beck AT, Steer RA, Garbin MG (1988). Psychometric Properties of the Beck Depression Inventory - 25 Years of Evaluation. Clin Psychol Rev.

[67] Mundt JC, Marks IM, Shear MK, Greist JH (2002). The Work and Social Adjustment Scale: a simple measure of impairment in functioning. Br J Psychiatry.

[68] Zahra D, Qureshi A, Henley W, Taylor R, Quinn C, Pooler J, Hardy G, Newbold A, Byng R (2014). The work and social adjustment scale: reliability, sensitivity and value. Int J Psychiatry Clin Pract.

[69] Attkisson CC, Zwick R (1982). The client satisfaction questionnaire. Psychometric properties and correlations with service utilization and psychotherapy outcome. Eval Program Plann.

[70] Larsen DL, Attkisson CC, Hargreaves WA, Nguyen TD (1979). Assessment of client/patient satisfaction: development of a general scale. Eval Program Plann.

[71] Shear MK, Brown TA, Barlow DH, Money R, Sholomskas DE, Woods SW, Gorman JM, Papp LA (1997). Multicenter collaborative panic disorder severity scale. Am J Psychiatry.

[72] Bohni MK, Spindler H, Arendt M, Hougaard E, Rosenberg NK (2009). A randomized study of massed three-week cognitive behavioural therapy schedule for panic disorder. Acta Psychiatr Scand.

[73] Baker SL, Heinrichs N, Kim HJ, Hofmann SG (2002). The liebowitz social anxiety scale as a self-report instrument: a preliminary psychometric analysis. Behav Res Ther.

[74] Chambless DL, Caputo GC, Jasin SE, Gracely EJ, Williams C (1985). The Mobility Inventory for Agoraphobia. Behav Res Ther.

[75] Bender DS, Morey LC, Skodol AE (2011). Toward a model for assessing level of personality functioning in DSM-5, part I: a review of theory and methods. J Pers Assess.

[76] Association AP (2013). Diagnostic and Statistical Manual of Mental Disorders (DSM-5\FFFD).

[77] Hutsebaut J, Feenstra DJ, Kamphuis JH (2016). Development and Preliminary Psychometric Evaluation of a Brief Self-Report Questionnaire for the Assessment of the DSM-5 level of Personality Functioning Scale: The LPFS Brief Form (LPFS-BF). Personal Disord.

[78] Hopwood CJ (2011). Personality traits in the DSM-5. J Pers Assess.

[79] Fonagy P, Luyten P, Moulton-Perkins A, Lee Y-W, Warren F, Howard S, Ghinai R, Fearon P, Lowyck B (2016). Development and Validation of a Self-Report Measure of Mentalizing: The Reflective Functioning Questionairre. PLoS One.

[80] Ha C, Sharp C, Ensink K, Fonagy P, Cirino P (2013). The measurement of reflective function in adolescents with and without borderline traits. J Adolesc.

[81] Krueger RF, Derringer J, Markon KE, Watson D, Skodol AE (2012). Initial construction of a maladaptive personality trait model and inventory for DSM-5. Psychol Med.

[82] Bach B, Maples-Keller JL, Bo S, Simonsen E (2016). The alternative DSM-5 personality disorder traits criterion: A comparative examination of three self-report forms in a Danish population. Personal Disord.

[83] Bo S, Bach B, Mortensen EL, Simonsen E (2016). Reliability and Hierarchical Structure of DSM-5 Pathological Traits in a Danish Mixed Sample. J Pers Disord.

[84] Gray MJ, Litz BT, Hsu JL, Lombardo TW (2004). Psychometric properties of the life events checklist. Assessment.

[85] Lund R, Christensen U, Nilsson CJ, Kriegbaum M, Hulvej Rod N (2014). Stressful social relations and mortality: a prospective cohort study. J Epidemiol Community Health.

[86] Lund R, Nielsen LS, Henriksen PW, Schmidt L, Avlund K, Christensen U (2014). Content validity and reliability of the Copenhagen social relations questionnaire. J Aging Health.

[87] Norman SB, Allard CB, Trim RS, Thorp SR, Behrooznia M, Masino TT, Stein MB (2013). Psychometrics of the Overall Anxiety Severity and Impairment Scale (OASIS) in a sample of women with and without trauma histories. Arch Womens Ment Health.

[88] Norman SB, Cissell SH, Means-Christensen AJ, Stein MB (2006). Development and validation of an Overall Anxiety Severity And Impairment Scale (OASIS). Depress Anxiety.

[89] Bentley KH, Gallagher MW, Carl JR, Barlow DH (2014). Development and validation of the Overall Depression Severity and Impairment Scale. Psychol Assess.

[90] Krogel J, Burlingame G, Chapman C, Renshaw T, Gleave R, Beecher M, Macnair-Semands R (2013). The Group Questionnaire: a clinical and empirically derived measure of group relationship. Psychother Res.

[91] Jones B, Jarvis P, Lewis JA, Ebbutt AF (1996). Trials to assess equivalence: the importance of rigorous methods. BMJ.

[92] Kazdin AE (2007). Mediators and mechanisms of change in psychotherapy research. Annu Rev Clin Psychol.

[93] Durham RC, Chambers JA, Power KG, Sharp DM, Macdonald RR, Major KA, Dow MG, Gumley AI (2005). Long-term outcome of cognitive behaviour therapy clinical trials in central Scotland. Health Technol Assess.

[94] Jakobsen JC, Hansen JL, Storebo OJ, Simonsen E, Gluud C (2011). The effects of cognitive therapy versus 'no intervention' for major depressive disorder. PLoS One.

